# The diagnostic role of albumin-based ratio markers for periprosthetic joint infection

**DOI:** 10.3389/fcimb.2026.1674043

**Published:** 2026-07-24

**Authors:** Panlong Fan, Qianqian Cao, Liya Sun, Ke Wang, Keli Guo, Yi Jin, Zhipeng Dai

**Affiliations:** 1 Department of Orthopaedics, Zhengzhou University People’s Hospital, Henan Provincial People’s Hospital, Zhengzhou, Henan, China; 2 Department of Cardiovascular Medicine, Affiliated Hospital of Shandong University of Traditional Chinese Medicine, Jinan, Shandong, China

**Keywords:** aseptic loosening, biomarkers, diagnosis, periprosthetic joint infection, serum albumin

## Abstract

**Background:**

Periprosthetic joint infection (PJI) is a serious complication following total joint arthroplasty. Timely and accurate diagnosis is crucial for the management of PJI. Numerous biomarkers are currently available for the diagnosis of PJI; however, a single indicator often fails to meet clinical requirements. In recent years, combinations of multiple biomarkers have demonstrated diagnostic potential in other infectious diseases. Nevertheless, the application of inflammatory marker combinations based on albumin (ALB) ratios in PJI diagnosis remains underexplored. Therefore, this study aims to investigate the diagnostic efficacy of novel biomarkers based on albumin ratios for PJI.

**Methods:**

A retrospective analysis was conducted on 257 patients with periprosthetic joint infection (PJI) and 137 with aseptic loosening (AL) who underwent revision arthroplasty at our institution between January 2019 and December 2024. We compared serum levels of C-reactive protein (CRP), erythrocyte sedimentation rate (ESR), albumin (ALB), the CRP/ALB ratio (CAR), the ESR/ALB ratio (EAR), and the composite index CAR + EAR (CEAR) between the two groups. Diagnostic performance was assessed using receiver operating characteristic (ROC) curves and the corresponding area under the ROC curve (AUC).

**Results:**

Patients with PJI exhibited significantly higher circulating levels of CRP, ESR, CAR, EAR and CEAR, whereas serum albumin was markedly lower than in the AL cohort. Subgroup analyses revealed no significant differences in the levels of these inflammatory biomarkers among the subgroups (P > 0.05). Based on ROC curve analysis, the novel inflammatory biomarker panels (CAR, EAR, CEAR) demonstrated high diagnostic accuracy: CAR yielded an AUC of 0.929 (95% CI: 0.905-0.953), EAR 0.890 (95% CI: 0.860-0.921), and CEAR 0.917 (95% CI: 0.890-0.943).

**Conclusion:**

Ratio-based markers based on ALB show promise as valuable new adjunctive diagnostic markers for PJI. The diagnostic efficacy of CEAR was comparable to that of CAR (AUC: 0.917 vs. 0.929, respectively), and both outperformed EAR (AUC = 0.890).

## 1 Introduction

Periprosthetic joint infection (PJI) is not only one of the most devastating complications after total joint arthroplasty (TJA) but also remains among the most formidable challenges in orthopedic surgery (Gundtoft et al., 2017). Evidence indicates that the anticipated surge in TJA volume through 2030 will inevitably yield a corresponding rise in the absolute incidence of PJI (Kurtz et al., 2007). PJI causes persistent joint dysfunction, substantially diminishes patients’ quality of life, and is independently associated with increased all-cause mortality, ultimately resulting in significantly compromised patient long-term clinical prognosis (Jafari et al., 2010; Koh et al., 2017; Xu et al., 2023). Consequently, timely and accurate identification of PJI is imperative for refining therapeutic strategies and improving patient prognosis (Zhang et al., 2024).

Although bacterial culture remains the clinical mainstay for the diagnosis of PJI, its prolonged turnaround time significantly impedes early detection (Denyer et al., 2023). In recent years, investigators have advocated novel techniques such as polymerase chain reaction (PCR), mass spectrometry, next-generation sequencing (NGS), and related molecular diagnostics for PJI diagnosis (Goswami et al., 2018; Esteban and Gómez-Barrena, 2021). Although these novel technologies markedly improve pre-operative PJI detection accuracy, their prohibitive costs and technical complexity impede deployment in resource-limited settings and primary-care clinics (Torchia et al., 2019). In contrast, peripheral blood tests are universally available, inexpensive and rapidly performed, making them attractive for both inpatient and ambulatory screening (Xu et al., 2022a). The traditional serological markers C-reactive protein (CRP) and erythrocyte sedimentation rate (ESR) have long been employed in PJI work-ups, yet their suboptimal specificity and susceptibility to confounding inflammatory conditions remain well-recognized shortcomings (Fernández-Sampedro et al., 2017). Consequently, there is a pressing need to identify novel, readily accessible biomarkers—either individually or as optimized composite panels—that can overcome the limitations of current diagnostic modalities.

Serum albumin, a well-established negative acute-phase reactant, serves as a key indicator reflecting both nutritional reserves and systemic inflammatory responses (Groznik et al., 2019). Its clinical significance has gained increasing recognition across various inflammatory disorders (Eckart et al., 2020). Recently, novel composite ratios based on inflammatory biomarkers—such as the C-reactive-protein-to-albumin ratio (CAR), platelet-to-lymphocyte ratio (PLR), and the C-reactive-protein-to-lymphocyte ratio (CLR)—have demonstrated significant clinical value in non-orthopedic inflammatory disorders (Utsumi et al., 2020; Wang et al., 2024; Albrecht et al., 2025). Moldovan et al. demonstrated that composite serum inflammatory markers, including the neutrophil-to-lymphocyte ratio (NLR), platelet-to-lymphocyte ratio (PLR), and monocyte-to-lymphocyte ratio (MLR), could serve as adjunctive diagnostic tools for PJI; however, these markers exhibited limited overall specificity and diagnostic performance (Moldovan, 2024). Klemt et al. further reported that whole blood composite inflammatory markers—namely PLR, the platelet count-to-mean platelet volume ratio (PVR), and NLR—possessed limited standalone diagnostic value and often required combination with ESR, CRP, and synovial fluid–related indices to facilitate PJI diagnosis (Klemt et al., 2022). In particular, Chen et al. reported that CAR correlates strongly with disease activity in inflammatory bowel disease (IBD) and outperforms conventional complete blood count parameters for discriminating active IBD (Chen et al., 2020). Concurrently, Shi et al. recently reported that CAR exhibited favorable diagnostic efficacy for PJI (AUC = 0.931; their study compared PJI patients against those with aseptic loosening), thereby highlighting the potential of albumin-related serum composite markers in PJI diagnosis (Shi et al., 2023). To date, however, the diagnostic role of the erythrocyte-sedimentation-rate-to-albumin ratio (EAR) in PJI remains unexplored. Therefore, this retrospective study is the first to systematically compare albumin-adjusted ratios (CAR, EAR, and their composite CEAR = CAR + EAR) against traditional inflammatory markers (CRP and ESR) for the diagnosis of PJI.

## 2 Materials and methods

### 2.1 Study design

This single-center, retrospective study consecutively enrolled all patients who underwent revision arthroplasty at our institution between January 2019 and December 2024. Cases that fulfilled the 2018 ICM criteria for periprosthetic joint infection (PJI) and those diagnosed with aseptic loosening (AL) were selected. Demographic variables (age, sex, surgical site) and pre-operative laboratory values—C-reactive protein (CRP), erythrocyte sedimentation rate (ESR) and serum albumin (ALB)—were extracted from the electronic medical record. From these data we calculated the ratios CRP/ALB (CAR), ESR/ALB (EAR) and the composite index CEAR (CAR + EAR). Intra-operative synovial fluid or purulent material obtained from every PJI patient was submitted for repeated aerobic and anaerobic cultures to serve as the microbiological reference standard. The diagnostic utility of each biomarker was then evaluated against these culture results. All blood samples were analyzed as part of routine clinical laboratory testing and were not processed in a single batch. However, all assays were performed using standardized automated analyzers with stringent quality control procedures, thereby minimizing the potential impact of inter-batch variability on the results. This study strictly adheres to the ethical principles of the Helsinki Declaration regarding human medical research and has been approved by the Ethics Committee of the People’s Hospital of Henan Province.

### 2.2 Definitions of PJI and AL

As shown in **Table 1**, the diagnostic criteria for PJI are based on the standards established by the Musculoskeletal Infection Society (MSIS) (Guan et al., 2019). The diagnosis of AL refers to the relevant criteria reported in previous literature, specifically including: (1) pain in the thigh or hip region, or knee pain; (2) radiographic evidence of prosthesis loosening, such as separation between the prosthesis components and bone tissue, displacement of the prosthesis components, or the presence of a radiolucent line; (3) negative periprosthetic culture; (4) exclusion of PJI (Huang et al., 2019).

**Table 1:** Musculoskeletal infection society criteria of the diagnosis of PJI.

Parameters	Number	MSIS criteria
Major criteria	1	Two positive periprosthetic cultures with phenotypically identical organisms
	2	There is a sinus tract that communicates to the joint
Minor criteria	1	Elevated serum CRP (>10 mg/l) or ESR (>30 mm/h)
	2	Elevated synovial fluid white blood cell count (>3000 cells/mL) or changed positive leukocyte esterase strip test (++ or +++)
	3	Elevated synovial fluid percentage of granulocytes (>80%)
	4	A single positive culture
	5	Changed positive histologic analysis of the periprosthetic tissue (>5 neutrophils in each of the 5 high-power fields at 400 × magnification)

PJI, periprosthetic joint infection; CRP, C-reactive protein; ESR, erythrocyte sedimentation rate; PJI is Defined by the criteria of ≥ 1 major or ≥ 3 minor criteria.

### 2.3 Inclusion and exclusion criteria

Inclusion criteria: (1) Patients diagnosed with PJI or AL and undergoing corresponding treatment. (2) All study indicators for the patients are complete and available.

Exclusion criteria were: (1) periprosthetic fracture; (2) prosthetic dislocation; (3) missing albumin data; (4) Patients with cancer; (5) albumin infusion therapy; (6) PJI occurring within 4 weeks after the initial total joint arthroplasty (Acute PJI triggers a robust acute-phase response, which can independently induce elevations in CRP and ESR as well as a reduction in ALB) (Workgroup on the Guidelines for the Diagnosis and Treatment of Prosthetic Joint Infection and Joint Surgery Committee of the Chinese Orthopaedic Association, 2021); (7) concomitant autoimmune disease (Autoimmune diseases are characterized by persistent chronic inflammation, which can sustain long-term abnormalities in CRP, ESR, and ALB levels);

### 2.4 Statistical analysis

All statistical analyses were performed using IBM SPSS Statistics 27.0 (IBM Corp., Armonk, NY, USA). Continuous variables with a normal distribution are presented as mean ± standard deviation (Mean ± SD). Non-normally distributed continuous variables were analyzed with the Mann–Whitney U test and expressed as quartiles. Categorical variables are presented as frequency (n) and percentage (%), and the chi-square test or Fisher’s exact test was selected according to data distribution. A P value < 0.05 was considered statistically significant. Receiver operating characteristic (ROC) curves, the area under the curve (AUC) and the 95% confidence interval (CI) were employed to assess the diagnostic value of each marker. The optimal cut-off value for each marker as a diagnostic tool for PJI was determined using the Youden Index. Subsequently, sensitivity, specificity, positive predictive value (PPV), and negative predictive value (NPV) were calculated to comprehensively evaluate the diagnostic performance of each marker for PJI. DeLong’s test was used to compare AUC differences between CRP vs. ESR, CRP vs. ALB, CRP vs. CAR, CRP vs. EAR, CRP vs. CEAR, ESR vs. CAR, ESR vs. EAR, and ESR vs. CEAR. A significance level of P < 0.05 indicated a statistically significant difference between indices. Statistical significance: *P<0.05, **P<0.01, ***P<0.001.

## 3 Result

### 3.1 Demographic data

**Figure 1:**
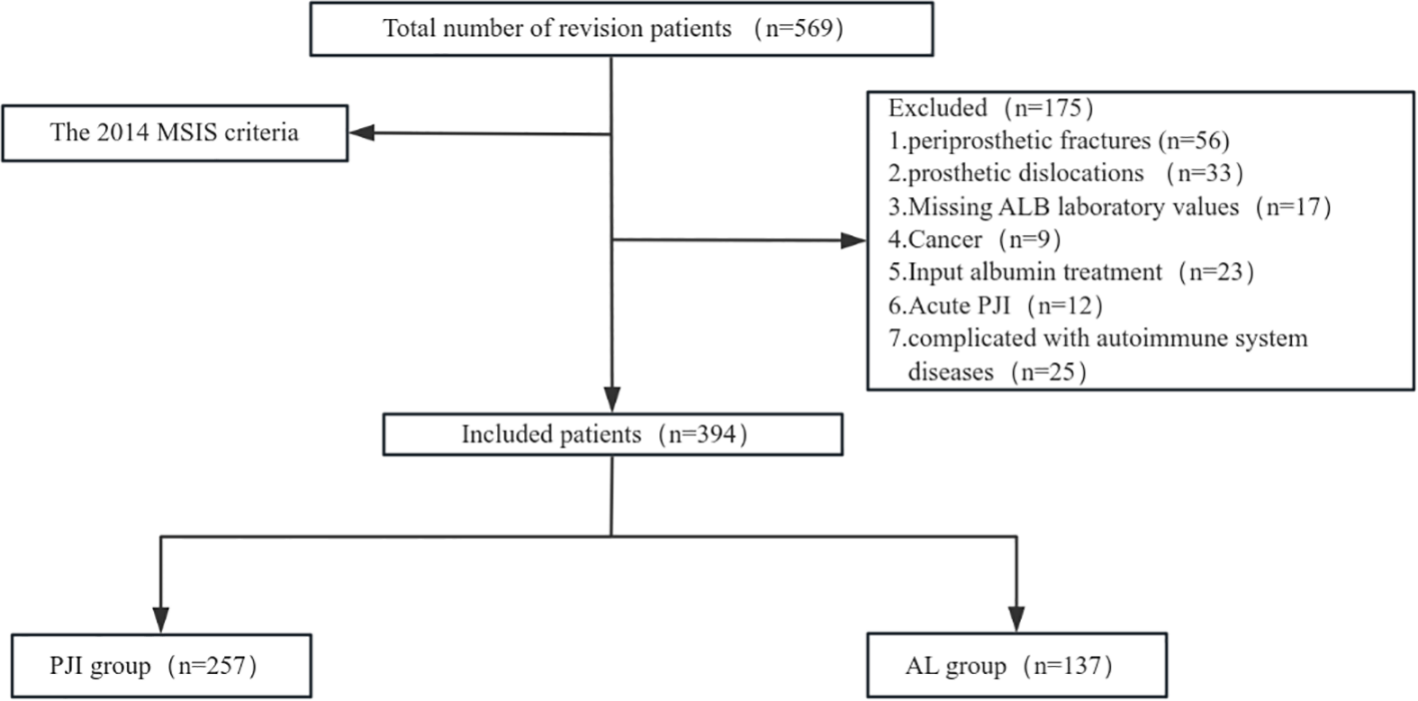
Flowchart of patient inclusion.

As illustrated in **Figure 1**, 569 patients who underwent revision knee or hip arthroplasty between January 2019 and December 2024 were initially screened. After applying the exclusion criteria, 175 patients were eliminated. Of the remaining 394 patients, 257 met the 2014 MSIS criteria for PJI and were assigned to the PJI group, whereas 137 patients were classified as AL. **Table 2** demonstrates that age and sex did not differ significantly between the two groups (P > 0.05). Further analysis revealed that the hip joint was more frequently affected in the AL group than in the PJI group (P < 0.001).

**Table 2:** Basic characteristics of the PJI group and the AL group.

Characteristics		Entire cohort (n=394)	PJI group (n=257)	AL group (n=137)	P value
Age^+^	66.08 ± 11.09		66.33 ± 11.20	65.59 ± 10.91	0.527
Gender^*^					
male	157(39.8)		104 (40.5)	53 (38.7)	0.731
female	237 (60.2)		153 (59.5)	84 (61.3)	
Joint^*^					
Hip	196 (49.7)		101 (39.3)	95 (69.3)	<0.001
Knee	198 (50.3)		156 (60.7)	42 (30.7)	

^+^ The values are given as the mean and standard deviation; ^*^The values are given in terms of number of cases and percentages. P<0.05 was considered statistically significant.

### 3.2 The levels of different markers in PJI group and AL group

**Figure 2:**
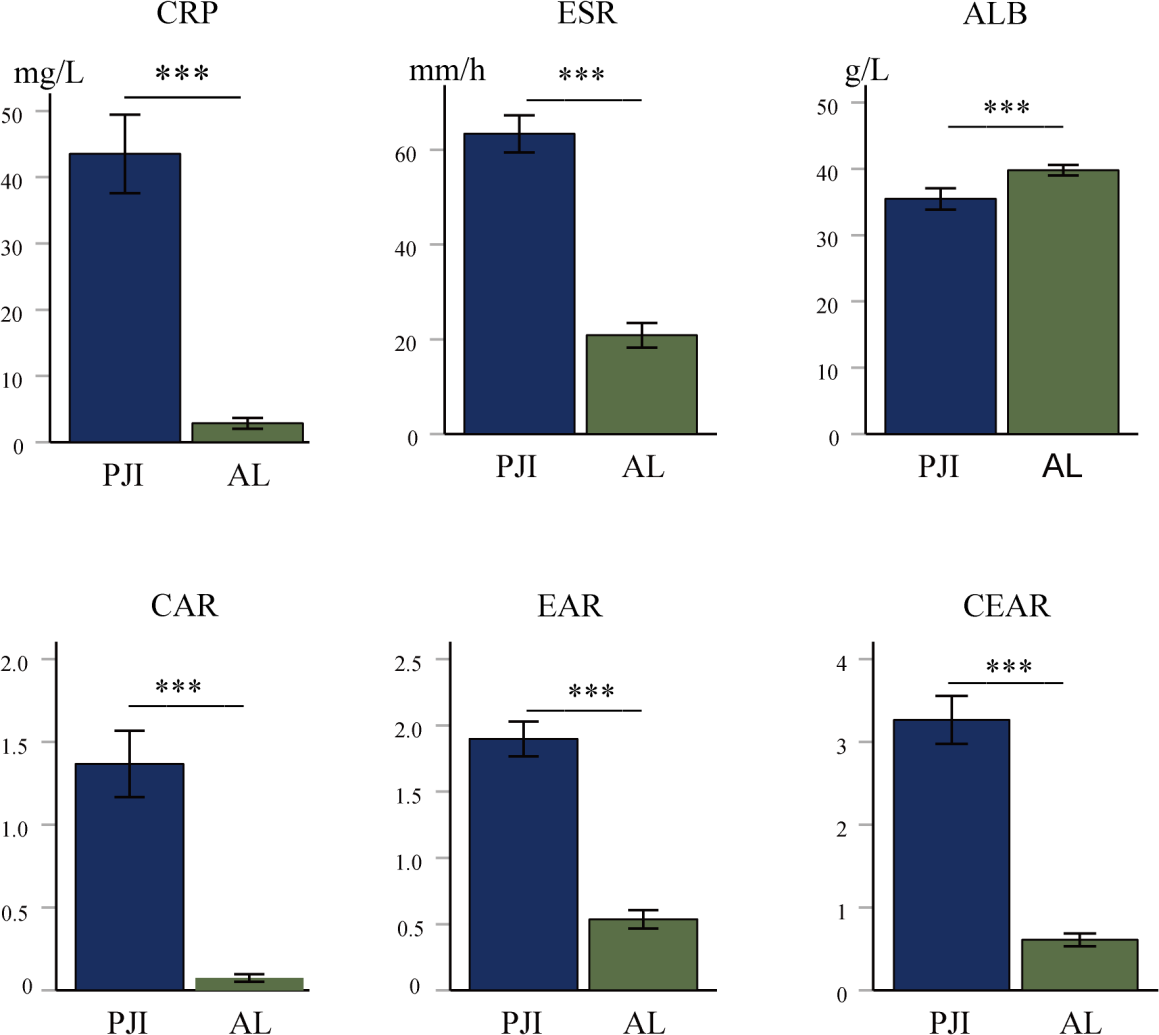
Comparison of levels of different markers between the PJI group and the AL. CRP, C-reactive protein; ESR, erythrocyte sedimentation rate; ALB, albumin; CAR, CRP/ALB; EAR, ESR/ALB; CEAR,CAR+CER. The symbol *** indicates statistical significance at the level of p < 0.001.

First, we compared the levels of traditional markers between the PJI and AL groups. The results showed that CRP (43.53 ± 48.25 vs 2.87 ± 4.90, P < 0.001) and ESR (63.41 ± 31.88 vs 20.84 ± 15.31, P < 0.001) were significantly higher, whereas ALB (35.47 ± 13.13 vs 39.79 ± 4.67, P < 0.001) was significantly lower in the PJI group than in the AL group (**Table 3**; **Figure 2**). Subsequently, we evaluated the albumin-based ratios. CAR (1.37 ± 1.63 vs 0.08 ± 0.14, P < 0.001), EAR (1.90 ± 1.07 vs 0.54 ± 0.41, P < 0.001) and CEAR (3.27 ± 2.36 vs 0.61 ± 0.45, P < 0.001) were all markedly elevated in the PJI group compared with the AL group (**Table 3**; **Figure 2**).

**Table 3:** Serologic markers of the PJI group and the AL group.

Markers	Entire cohort (n=394)	PJI group (n=257)	AL group (n=137)	P value
Markers				
CRP (mg/L)	29.39 ± 43.60	43.53 ± 48.25	2.87 ± 4.90	<0.001
ESR (mm/h)	48.61 ± 33.99	63.41 ± 31.88	20.84 ± 15.31	<0.001
ALB (g/L)	36.97 ± 11.14	35.47 ± 13.13	39.79 ± 4.67	<0.001
CAR	0.92 ± 1.45	1.37 ± 1.63	0.08 ± 0.14	<0.001
EAR	1.42 ± 1.11	1.90 ± 1.07	0.54 ± 0.41	<0.001
CEAR	2.34 ± 2.30	3.27 ± 2.36	0.61 ± 0.45	<0.001

The values are given as the mean and standard deviation;CRP, C-reactive protein; ESR, erythrocyte sedimentation rate; ALB, albumin; CAR, CRP/ALB; EAR, ESR/ALB; CEAR,CAR+CER. Statistical significance: P<0.05.

### 3.3 Culture results of patients in the PJI group

To evaluate the diagnostic utility of the aforementioned biomarkers, the study included 207 PJI patients with positive cultures and 50 with negative cultures (**Table 4**). Among culture-positive cases, *Staphylococcus aureus* (n = 52) and *Staphylococcus epidermidis* (n = 48) were the predominant pathogens (**Table 4**). When these two major pathogen groups were compared, no statistically significant differences were observed in any biomarker level (CRP, ESR, ALB, CAR, EAR, or CEAR) (P > 0.05), indicating that the biomarker results were not significantly influenced by the infecting pathogen species (**Table 5**).

**Table 4:** Different culture results of PJI group.

Culture organism	No. of patients
Positive	207
Staphylococcus aureus	52
Staphylococcus epidermidis	48
Colibacillus	21
Streptococcus	9
Staphylococcus hemolyticus	6
Pseudomonas aeruginosa	5
Klebsiella pneumoniae	5
Fungus	17
Other	44
Negative	50

**Table 5:** Comparison of levels of different markers between the *S.aureus* group and the *S.epidermidis* group.

Markers	*S. aureus* (n=52)	*S. epidermidis* (n=48)	P value
Markers			
CRP (mg/L)	44.38 ± 25.87	43.04 ± 20.01	0.774
ESR (mm/h)	64.69 ± 23.57	61.92 ± 24.48	0.565
ALB (g/L)	36.74 ± 27.71	35.90 ± 4.26	0.837
CAR	1.39 ± 0.84	1.22 ± 0.58	0.243
EAR	2.05 ± 1.03	1.75 ± 0.72	0.096
CEAR	3.44 ± 1.59	2.97 ± 1.12	0.093

CRP, C-reactive protein; ESR, erythrocyte sedimentation rate; ALB, albumin; CAR, CRP/ALB; EAR, ESR/ALB; CEAR, CAR+CER. *S. aureus, Staphylococcus aureus; S. epidermidis, Staphylococcus epidermidis*.

### 3.4 The diagnostic value of different markers

**Figure 3:**
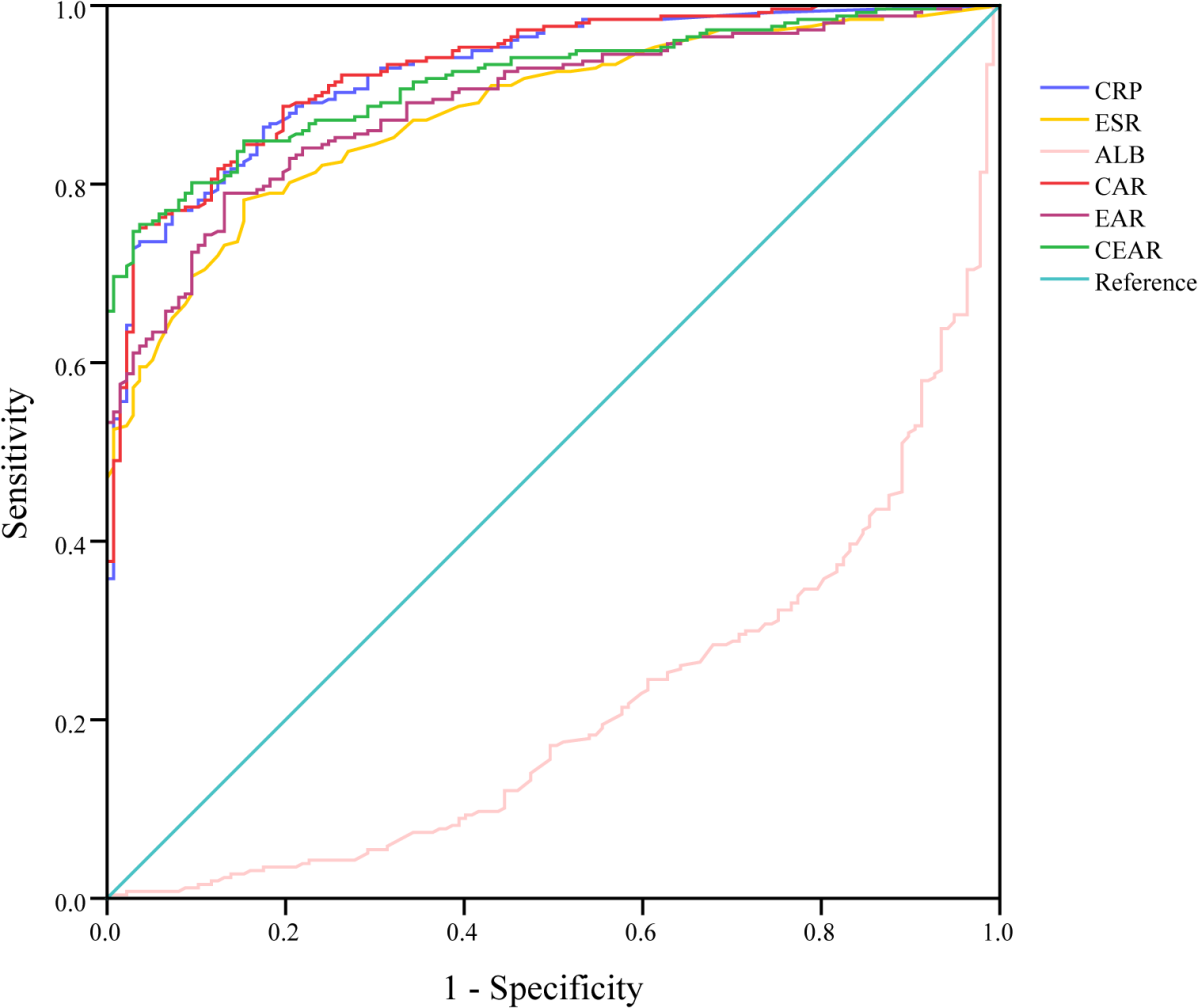
The ROC curves of CRP, ESR, ALB, CAR, EAR and CEAR. CRP, C-reactive protein; ESR, erythrocyte sedimentation rate; ALB, albumin; CAR, CRP/ALB; EAR, ESR/ALB; CEAR,CAR+CER.

ROC-curve analysis indicated that CRP (AUC = 0.926, 95% CI 0.901–0.950), CAR (AUC = 0.929, 95% CI 0.905–0.953) and CEAR (AUC = 0.917, 95% CI 0.890–0.943) exhibited “excellent” discriminatory power (AUC > 0.9), whereas ESR (AUC = 0.880, 95% CI 0.848–0.912) and EAR (AUC = 0.890, 95% CI 0.860–0.921) were classified as “good” (0.80 < AUC < 0.89); ALB showed poor performance (AUC = 0.214, 95% CI 0.168–0.260) (**Table 6**, **Figure 3**). Among all markers, CAR achieved the highest AUC of 0.929 (95% CI 0.905–0.953), enabling differentiation of PJI from AL at an optimal cut-off of 0.232 with 74.7% sensitivity and 97.1% specificity. Notably, both CAR and EAR demonstrated enhanced diagnostic capacity compared with CRP and ESR, respectively (CAR vs CRP: 0.929 vs 0.926; EAR vs ESR: 0.890 vs 0.880). CEAR also showed excellent diagnostic utility, delivering 75.5% sensitivity and 96.3% specificity at its optimal cut-off of 1.535 (**Table 6**; **Figure 3**). Based on the Youden index, the predictive values were calculated as follows: CRP (PPV 94.42%, NPV 66.00%), ESR (PPV 90.54%, NPV 67.44%), ALB (PPV 45.63%, NPV 13.30%), CAR (PPV 97.95%, NPV 66.83%), EAR (PPV 91.86%, NPV 68.79%) and CEAR (PPV 97.49%, NPV 67.69%), underscoring the high reliability of CAR, EAR and CEAR (**Table 6**; **Figure 3**).

**Table 6:** The diagnostic performance of different markers for PJI.

Markers	AUC	95% CI	Youden index	Optimal cutoff value	Sen	Spec	PPV (%)	NPV (%)
CRP (mg/L)	0.926	(0.901, 0.950)	0.699	9.11	0.735	0.964	94.42	66.00
ESR (mm/h)	0.880	(0.848, 0.912)	0.629	36.5	0.782	0.847	90.54	67.44
ALB (g/L)	0.214	(0.168, 0.260)	0.452	36.755	0.366	0.182	45.63	13.30
CAR	0.929	(0.905, 0.953)	0.718	0.232	0.747	0.971	97.95	66.83
EAR	0.890	(0.860, 0.921)	0.658	0.958	0.790	0.869	91.86	68.79
CEAR	0.917	(0.890, 0.943)	0.718	1.535	0.755	0.963	97.49	67.69

CI, confidence interval; CRP, C-reactive protein; ESR, erythrocyte sedimentation rate; ALB, albumin; CAR, CRP/ALB; EAR, ESR/ALB; CEAR, CAR+CER; Sen, Sensitivity; Spec, Specificity; PPV, Positive Predictive Value; NPV, Negative Predictive Value.

DeLong analysis further revealed that CAR performed marginally better than CRP (Z = -1.950, P = 0.051) and was significantly superior to ESR (Z=–3.178, P = 0.001) and ALB (Z= -24.055, P < 0.001). No significant difference was observed between CAR and CEAR (Z = 1.182, P = 0.237); however, both CAR and CEAR significantly outperformed EAR (CAR vs EAR: Z = 2.654, P = 0.008; CEAR vs EAR: Z = -4.462, P < 0.001) (**Table 7**).

**Table 7:** Pairwise comparison of ROC curves among CRP, ESR, ALB, CAR, EAR and CEAR in PJI diagnosis.

Variable	CRP	ESR	ALB	CAR	EAR	CEAR
CRP	–	Z=2.958 p = .003	Z=24.406 p <.001	Z= -1.950 p = .051	Z=2.367 p = .018	Z=0.828 p = .408
ESR		–	Z=21.530 p <.001	Z= -3.178 p = .001	Z= -2.349 p= .019	Z= -4.654 p <.001
ALB		–	–	Z= -24.055 p <.001	Z= -21.057 p <.001	Z= -23.006 p <.001
CAR				–	Z= 2.654 p = .008	Z= 1.182 p = .237
EAR					–	Z= -4.462 p <.001
CEAR						–

CRP, C-reactive protein; ESR, erythrocyte sedimentation rate; ALB, albumin; CAR, CRP/ALB; EAR, ESR/ALB; CEAR,CAR+CER. A dash (-) indicates comparisons of a variable with itself. Bolded p values indicate statistical significance (p <.05).

## 4 Discussion

PJI represents a catastrophic complication after total joint arthroplasty, and distinguishing it from AL is pivotal for therapeutic decision-making. Although CRP and ESR are widely used because of their high sensitivity and low false-negative rates, their limited specificity markedly restricts diagnostic efficacy. Therefore, identifying novel biomarkers capable of early and precise diagnosis remains imperative. Accumulating evidence indicates that ALB holds diagnostic potential in a spectrum of inflammatory disorders (Cao et al., 2022; Sheng et al., 2022; Yang et al., 2023). In this context, the present study is the first to systematically evaluate the diagnostic utility of albumin-based composite ratios in PJI. Subgroup analyses according to bacterial culture results revealed no statistically significant differences in the levels of these inflammatory indices under different pathogen conditions (P > 0.05). Compared with traditional markers (CRP and ESR), the newly developed ratio biomarkers CAR, EAR, and their integrated metric CEAR demonstrated superior diagnostic performance. Collectively, albumin-based composite ratios (CAR/EAR/CEAR) may serve as promising adjunctive tools for PJI diagnosis.

CRP, an acute-phase protein synthesized by the liver, rises markedly in response to inflammatory stimuli and serves as a pivotal biomarker of systemic inflammation (Parvizi et al., 2012). Although CRP demonstrates diagnostic utility for PJI, its diagnostic accuracy remains constrained (Sigmund et al., 2021). Previous studies indicate that CRP is insufficient for identifying low-virulence pathogens and chronic PJI (Pérez-Prieto et al., 2017). and serum CRP–based criteria are prone to false-negative results (Wang et al., 2021). Notably, Xu et al. demonstrated that combining CRP with fibrinogen (FIB) enhances diagnostic accuracy for PJI (Xu et al., 2022b). and multiple independent investigations have confirmed that CRP-based composite indices outperform CRP alone (Balta et al., 2023; Wu et al., 2023; Yu et al., 2023). ALB, another hepatically derived protein, declines markedly during infection or inflammation secondary to impaired synthesis, increased vascular leakage, and accelerated catabolism (Dirajlal-Fargo et al., 2018). Although ALB has traditionally been used to assess nutritional status and predict prognosis (Mukai et al., 2018; Jin et al., 2022). its concentration inversely correlates with inflammatory burden (Ji et al., 2023). and isolated ALB measurements exhibit limited diagnostic value (Liu et al., 2025). Intriguingly, the inverse relationship between rising CRP and falling ALB during infection has attracted considerable attention, positioning the CAR as a promising inflammatory marker (Haider Kazmi et al., 2022). CAR has been widely adopted to diagnose inflammatory disorders and to stratify disease severity,often surpassing the performance of conventional biomarkers such as CRP and ESR (Seringec Akkececi et al., 2019; Katkat et al., 2022). For instance,it independently predicts the severity of acute cholecystitis (Sato et al., 2018). and differentiates complicated from uncomplicated paediatric appendicitis in pediatric populations (Hou et al., 2022). Choe et al. reported that CAR achieves an AUC of 0.97 for PJI diagnosis (Zhang et al., 2022). Therefore, the present study enrolled a larger cohort of PJI and AL patients to validate these findings. Our results confirmed that CAR demonstrates excellent discriminatory ability (AUC = 0.929, 95% CI 0.905–0.953). At the optimal cut-off of 0.232, CAR exhibited 74.7% sensitivity, 97.1% specificity, 97.95% PPV and 66.83% NPV. Collectively, CAR holds promise as a routine biomarker for PJI diagnosis.

ESR reflects the velocity at which red blood cells settle under standardised conditions and is markedly elevated in inflammatory diseases (Maier et al., 2020). It has been adopted as a first-line screening marker for PJI; however, a key limitation is its susceptibility to elevation in diverse systemic inflammatory conditions (Shahi et al., 2015). Previous work has shown that the ESR/CRP ratio can help distinguish infection from disease activity in febrile patients with systemic lupus erythematosus (Corsonello et al., 2011). Building on this, we further investigated the diagnostic performance of EAR as a novel inflammatory biomarker. ESR accelerates with elevated positive acute-phase reactants (e.g., fibrinogen), while ALB decreases significantly during inflammation due to suppressed hepatic synthesis and increased catabolic breakdown. This combination produces a synergistic amplification effect in EAR, which better captures inflammation-related physiological changes and reduces interference from confounding factors (Ding and Xue, 2022; Kaplan et al., 2023). Studies have also shown that ESR exhibits circadian variation, and incorporating albumin into EAR may partially correct such non-inflammatory fluctuations (Yucel et al., 2021). In addition, albumin-based adjustment can mitigate the influence of inter-individual differences in baseline nutritional status on absolute ESR values, thereby improving assay stability (Zahedi et al., 2023).However, no studies to date have explored the role of EAR in this disease setting. Accordingly, we further investigated the diagnostic value of EAR in PJI. In the present cohort, EAR demonstrated good discriminatory performance (AUC = 0.890), suggesting that it is a potentially superior alternative to isolated ESR for PJI diagnosis.

Multiple investigations have reported that combining biomarkers enhances diagnostic accuracy for PJI (Jiao et al., 2022; Choe et al., 2023). Here, both CAR (AUC = 0.929) and EAR (AUC = 0.890) exhibited strong individual performance.Capitalising on these complementary strengths, we developed an integrated composite index, CEAR. CEAR achieved an excellent AUC of 0.917 and, at the optimal cut-off of 1.535, provided 75.5% sensitivity and 96.3% specificity for distinguishing PJI from aseptic loosening, confirming the additive value of albumin-based composite indices in PJI diagnostics. In the present study, CAR and CEAR demonstrated high specificity but only moderate sensitivity. The specificities reached 97.1% and 96.3%, respectively, with excellent positive predictive values, making them suitable for confirming a diagnosis of PJI. However, their sensitivities were only 74.7% and 75.5%, indicating limited ability to rule out PJI based on negative results; thus, they are not suitable for standalone screening. Therefore, in clinical practice, we recommend combining CAR and CEAR with highly sensitive conventional markers such as CRP and ESR to optimize the screening and diagnostic workflow for PJI.

## 5 Limitations

This study systematically assessed the diagnostic potential of albumin-based biomarkers (CAR, EAR, and CEAR) for PJI. By comparing these novel indices with traditional markers such as CRP and ESR, we obtained a comprehensive understanding of their relative efficacy and provided clinically relevant insights. Nevertheless, several limitations should be acknowledged: (1) the retrospective design inherently carries selection bias; (2) the single-centre setting and limited sample size necessitate validation in larger, multicentre cohorts; and (3) The subgroup analysis of this study (*Staphylococcus aureus* vs. *Staphylococcus epidermidis*) was limited by a small sample size. Although no formal *post-hoc* power analysis was performed, the limited sample size suggests the statistical power may be insufficient. Thus, negative findings should be interpreted with caution, and future studies with larger sample sizes are needed to further validate these results. Therefore, prospective multicentre studies with larger populations are warranted to confirm our findings.

## 6 Conclusion

This study demonstrates that, compared with traditional markers such as CRP and ESR, the albumin-based ratios CAR, EAR, and their composite index CEAR confer significantly higher diagnostic value for PJI. Among these markers, CAR and CEAR yielded similarly high diagnostic performance, yet CEAR did not outperform CAR. This indicates that combining EAR with CAR provides no additional diagnostic benefits. EAR also showed acceptable efficacy when used alone, albeit slightly lower than CAR and CEAR. Given their favorable diagnostic performance and easy clinical applicability, CAR, EAR and the combined marker CEAR are promising candidate biomarkers. Nevertheless, further studies are required to validate their application in routine clinical practice.

## Data Availability

The original contributions presented in the study are included in the article/supplementary material. Further inquiries can be directed to the corresponding authors.
